# The Influences of Drivers/Riders in Road Traffic Crashes in Ghana between 2001 and 2011

**DOI:** 10.5539/gjhs.v6n4p49

**Published:** 2014-04-07

**Authors:** Thompson Amo

**Affiliations:** 1 Public Health Management, Graduate School of Asia Pacific Studies, Ritsumeikan Asia Pacific University, Beppu, Japan

**Keywords:** road traffic accident, drivers/riders, death, injury, Ghana

## Abstract

The road traffic accident (RTA) is a global misfortune and the leading cause of death among young drivers. In safeguarding and developing innovative safety strategies to curtail the situation, the factors causing this menace needs proper attention and investigation. The objective of this study is to identify the potential factors responsible for causing a traffic accident in Ghana. In studying these factors extensively, a descriptive study with quantitative technique was employed. Analyses used data between 2001 and 2011 obtained from the Building and Road Research Institute (BRRI) with specific focus on the age, drinking, vehicle defect, driver/rider error, injury, road surface type and weather. A total of 200,528 cases of drivers/riders were analysed and discovered that, people with younger age (21-40) contribute 62.97% of total crashes. Crashes reduce steadily as drivers/riders age increases. Also, the vehicle defect analysis shows that 87.46% of accidents cannot be linked to the fault of the vehicle before incidence, while the majority (75.38%) of drivers/riders had no injury during a traffic accident. Higher number of fatalities are recorded on tar good roads (81.57%) and clear weather (91.75%). The fight against this canker by the authorities must consider periodic refresher courses for younger drivers/riders on traffic law to bring to bear the adherence of good driving/riding principles and attitudes to ensure that safety is guaranteed for all road users in the country.

## 1. Introduction

It is an undeniable and indisputable fact that road traffic accidents are a tragedy and a great ordeal for national development, especially in less developed Nations. The road traffic injury is a global phenomenon and a leading cause of death among young drivers of cars or ride motorized two-wheelers. The rate of death is higher, particularly among teenagers and males. A case-control study, according to the World Health Organization (WHO) report in 2009 established that one third of all traffic accidents comprising young drivers should have been controlled if they had been restricted to driving with no more than one passenger (WHO, 2009).

Another study by National Research Council (NRC) et al. (2007), depicts that crashes of automobiles, are major causes of deaths of people between 4 and 34 years old. The rate is highest for new drivers during the first few months driving on their own. The statistics show that the likelihood of new or the first six months of solo driving involved in the fatal accident is 8 times higher than older or more experienced drivers. The teenagers are more susceptible to the risks of drinking and driving. The younger and freshly licensed drivers, have reasonably limited alcohol-related crashes, while older and more experienced teenagers and young adult drivers have more of aforementioned crashes than adults do.

The African region continues to be the leading cause of traffic fatalities but the least motorized compared to the other five regions. It possesses only 2% of the world’s vehicles, but has accounted for 16% of the global deaths (WHO, 2013). Ghana as a developing country in the African region has a fair share in contributing factors to this unfortunate predicament. A Common knowledge holds that a specific age and gender categories are more vulnerable and likely to be at fault when a traffic accident occurs due to inexperience and lifestyle behaviour. It is believed that drivers of younger ages are felt to be at fault due to high-risk lifestyles (Gregerson & Berg, 1994), the tendency of unreasonably enthusiastic judgments of driving ability or accident risk (DeJoy, 1992) and dangerous driving errors and violations (Blockley & Hartley, 1995). Driving involves both a level of physical coordination and an ability to make judgments which in most cases are taken for granted. It requires both the ability to determine and physical coordination of the person involved at any point in time. The skill level is vital and to become a good driver depends on the essential improvement of the design of the road (Karl et al., 1998).

In Ghana, the deaths and the occurrences of road traffic accidents are growing even though efforts are ongoing to reduce it to a single digit by the year 2015. It is estimated to cost the country $418 million converted to be 1.6% of Gross Domestic Product (GDP) (National Road Safety Commission (NRSC), 2012 & WHO, 2013). The government liberalization policy of safeguarding the availability of vehicles has greatly increased the number of vehicles in the country. In the year 2000, a total number of duly registered vehicles were 511,062 but rose to 1,122,700 in 2010 (NRSC, 2010). It is expected that the rising trend in the number of vehicles on the road should correspond with road maintenance, driver education, vehicle’s upkeep and traffic enforcement. But unfortunately, less has been achieved in this regard. The result is that the roads have become death traps.

The operational standards of many drivers considered to be poor poses a serious impact on the problem of traffic accidents. A combination of factors such as lack of education of many drivers coupled with extensively low economic development in general are identified (Sarpong, 2010). The Driver Vehicle and Licensing Authority (DVLA) and other stakeholders in the transport industry have put the problem into focus and are seeking answers to improve the current conditions. However, the growing trends and daily life loss raise questions about whether or not these cankers are triggered by the weather condition, the driver/riders action, the vehicle, or the road itself. In safeguarding and developing safety strategies, the contributing factors to this need further investigation.

This research takes into accounts analysis of influences of drivers/riders in RTA in Ghana between 2001 and 2011. This research is unique and the first of its kind to be conducted in the entire country. It is believed that the results would inform stakeholders what factors might contribute to this predicament in order to develop an innovative and effective means of ensuring road safety. This would be helpful in efficiently managing and making a productive use of the limited resources used in fighting against RTA in the country.

## 2. Materials and Methods

A descriptive study based on quantitative approach was adopted to explore the influences of drivers/riders in RTA in more depth. The Building and Road Research Institute (BRRI) under the umbrella of the Council for Scientific and Industrial Research (CSIR) in collaboration with the Motto Traffic and Transport Unit (MTTU) of the Ghana Police Service crashes data from 1st January, 2001 to 31st December, 2011 were used for the investigation. This data is compiled by BRRI from Police records on reported traffic accident cases using a standard report form. The information on this form have about 69 variables related to year, casualties, accident severity and location, the vehicles involved and so on. Evidence to authenticate data accuracy show that the WHO, National Road Safety Commission and Ministry of Transport; Ghana, uses the same dataset for annual reports regarding traffic accident. Parameter estimate for the purpose of this research take into accounts some variables with its respective categorizations such as driver/rider age (from 1-100, unknown), injury (fatal, hospital, injury, no injury, unknown), driver/rider error (none, inexperience, inattentive, too fast, too close, no signal, improper overtaken, fatigue/asleep, loss of control, other), drinking and driving (not suspected, suspected, tested and positive, tested and negative, unknown), vehicle defect (none, brakes, steering, tyres, suspension, rollbars, multiple, light, other), road surface type (tar good, tar few potholes, tar many potholes, gravel, earth few potholes, earth many potholes, unknown) and weather/visibility (clear, fog, rain, dusty, dazzle, other). The absolute numbers were converted into percentages and figures generated to represent individual parameters selected using Microsoft excel 2010.

## 3. Results

**Figure 1:**
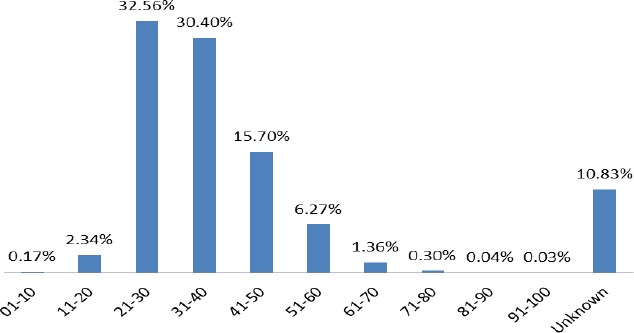
The age distribution of drivers/riders involved in crashes from 2001-2011

Figure 1 presents information on the drivers/riders involved in traffic accidents between 2001 and 2011. There is 10 years difference in the age categorization starting from 01 to 100. In order to evaluate the individual age responsibility for crashes, percentages were obtained for each category. It can be observed that young drivers are more likely to be involved in a traffic accident. Among the age categories, people between 21-30 years alone contribute 32.56% of RTA. The ages between 21 and 60 contribute 84.93% of fatalities. The opposite holds as older drivers/riders (61-100) is 1.73%. The “unknown” according to BRRI are cases that could not be determined to identify the specific age group they belong, contributed 10.83%. This means that the rate of crashes reduces as the driver/rider age increases.

**Figure 2:**
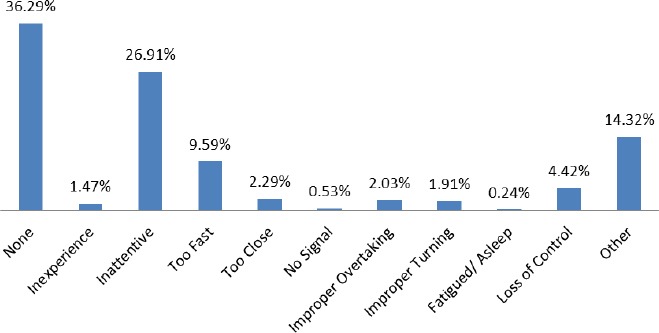
The distribution of driver/rider error from 2001-2011

Figure 2 shows the statistics of errors committed by drivers/riders at the time of the traffic accident. The categorization in respect of these is: none, inexperience, inattentive, too fast, too close, no signal, improper overtaking, improper turning, fatigued/asleep, loss control and other. The percentage of these categories are 36.29%, 1.47%, 26.91%, 9.59%, 2.29%, 0.53%, 2.03%, 1.91% and 0.24% respectively. This shows 48.86% of the traffic accident within this period could be attributed to the fault of the drivers/riders.

**Figure 3:**
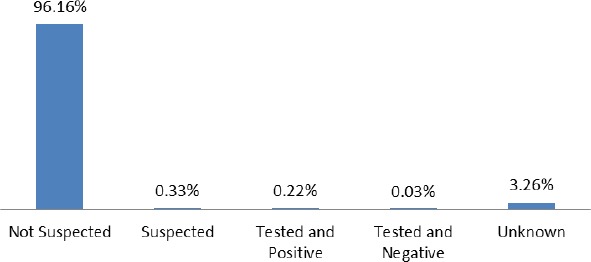
The distribution of drinking and driving/riding from 2001-2011

Figure 3 presents the test result of drivers/riders after crashes has happened. The categories are stated as: not suspected, suspected, tested and positive, tested and negative and unknown. The highest of all is the not suspected with a percentage of 96.16%. This is followed by the unknown (3.26%) who is supposedly drivers/riders who were not classified during data collection. Those suspected were 0.33%, tested and positive was 0.22%, while those tested but found to be negative is 0.02%. This suggests that, majority of drivers in RTA is not suspected in drinking alcohol.

**Figure 4:**
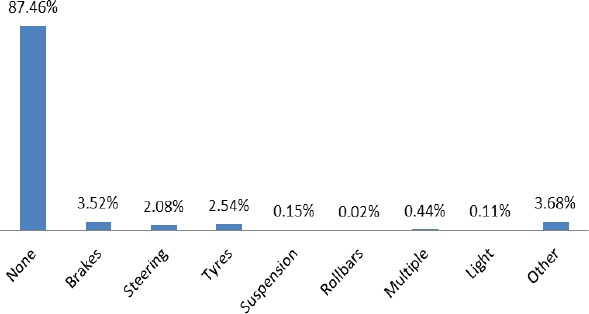
The distribution of vehicle defect from 2001-2011

Figure 4 shows information on defects of all vehicles involved in traffic accident. The vehicle defect is categorized into none, brakes, steering, tires, suspension, roll bars, multiple, light and other. The data is calculated to give a clear picture of each category contribution of this unfortunate event of the road. The percentage for each category are 87.46%, 3.52%, 2.08%, 2.54%, 0.15%, 0.02%, 0.44%, 0.11% and 3.68% respectively. This shows that, none (87.46%) of the vehicles proved to have no defect before the occurrence of RTA. All other factors accounts for 12.54% of total RTA within the period of investigation.

**Figure 5:**
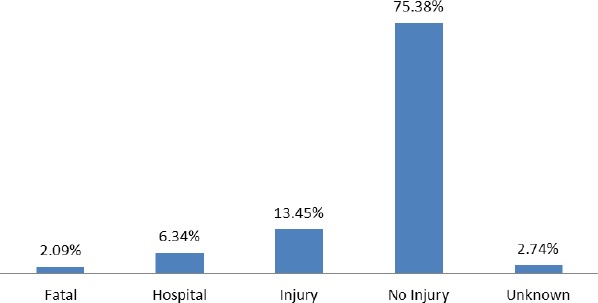
The distribution of driver/rider injury from 2001-2011

Figure 5 discusses the distribution of driver/rider injuries sustained during a traffic accident. The categories for the distribution are fatal, hospital, injury, no injury and unknown. The statistics show that the majority of drivers/riders (75.38%) did not sustain any injury. 13.45%, 6.34%, 2.74% and 2.09%, respectively, represent drivers/riders who had an injury, hospital, unknown and fatal crash cases.

**Figure 6:**
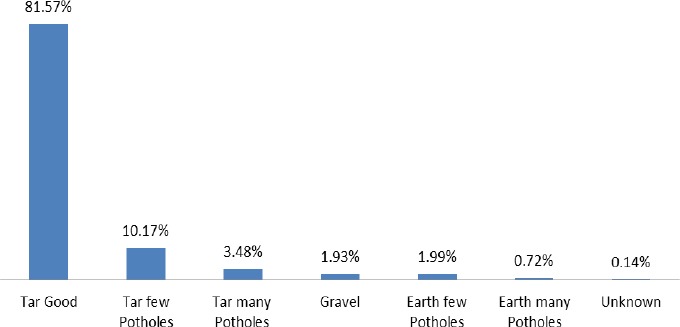
Road surface type

The statistics on the road surface type in Figure 6 show that tar road considered to be good (81.57%) was where accidents occurred most. Tar with few potholes (10.17%), tar many potholes (3.48%), earth with few potholes (1.99%), gravel (1.93%), earth with many potholes (0.72%) and unknown (0.14%) in that order represent percentages recorded according to the number of recorded traffic cases with regards to road surface type between 2001 and 2011.

**Figure 7:**
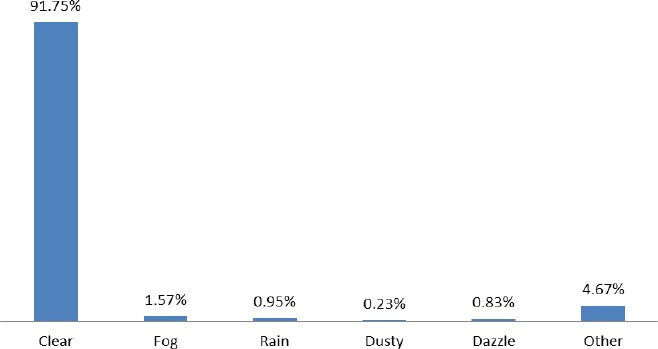
Weather/Visibility

Figure 7 presents the visibility recorded at the time of the incident. The categories are made up of clear, fog, rain, dusty, dazzle and other. The highest among them is clear (91.75) followed by other (4.67%) fog (1.57%), rain (0.95%), dazzle (0.83%) and dusty (0.23%).

## 4. Discussion

This study discusses the influences of drivers/riders in traffic accident cases from 1st January, 2001 to 31st December, 2011. Analysis focused on parameters such as age, drinking, vehicle defect, driver/rider error, injury sustained, road surface type and weather condition during a traffic accident. A total of 200,528 traffic accident cases of drivers/riders was analysed and presented in figures for easy understanding of results. The results show that there are differences in the crashes distribution with drivers/riders age. People with younger age (21-40) contribute 62.96% of total crashes. Crashes reduce steadily as drivers/riders age increases. Also, crashes are at its peak between ages of 21 to 30 (32.56%). This results support findings by (Gregerson & Berg, 1994), which says that specific age and gender categories are more likely to be at fault. They are unreasonably enthusiastic judgments of driving ability or accident risk (DeJoy, 1992), and dangerous driving errors and violations (Blockley & Hartley, 1995). The high number of young people involving in causalities could be attributed to the general youthful structure of the population in the country.

The test result of drivers/riders after crashes have occurred shows that 96.16% of them were not suspected for taken any alcoholic beverage. This result contradicts the observation made by (Gerald et al., 1999), which shows that one-quarter of drivers were suspected of driving under the influence of alcohol, and (Charlton & Smith, 2003), that alcohol is one of the main threat to road safety. This could be linked to the enforcement of Road Traffic Act, 2004 (Act 683), and its Regulations, 1974, which imposes stiffer punishment to culprits. The law stipulates that, drinking of any alcoholic substance during driving, either in a form of beverage or high drugs when proven by a court of competent jurisdiction, a fine of GH¢ 2,400 (USD$ 1,264, March 2013, Bank of Ghana statistical bulletin) or may be sentenced up to 3.3 years’ imprisonment or both. However, 0.22% of drivers/riders tested showed a positive sign of alcohol intake. The authorities must not be adamant and relent in their efforts, but make sure that those who indulge in such activities are not allowed to drive and have their license ceased permanently.

The analysis of driver/rider error was deemed important because it shows whether or not the driver/rider must be blamed for the cause of the accident. The analyses show that 63.71% of the causes of the traffic accident were as a result of driver/rider error. The most outstanding among them was inattentive (26.91%) on the part of drivers/riders. Inattentive could be attributed to the use of mobile phone, tiredness as a result of loss of energy, engaging in conversations with passengers and the like. This result confirms (Mondal et al., 2011), that three-fourth of the accidents were as a result of drivers fault. This is followed by other (14.32%) and 4.42% being loss of control. All these possibilities must be looked at when sensitizing drivers in avoiding road accident. The Motto Traffic and Transport Unit (MTTU) should lay emphasis on speed limits of vehicle on the road which has a potential of preventing and lessening the impact of severity of the injuries and casualties.

Findings on the distribution of vehicle defect show that a greater percentage (87.46%) of traffic accidents cannot be linked to the technical deficiency of the vehicle before the incident. This reaffirms the fact the cause of the accident is human error (63.71%). All other defects such as brakes, steering tires, suspension, roll bars, multiple, light and others combine to give 12.54%, which is significant and ought to pay attention to in safety tragedies on the roads to reduce deaths and disabilities.

The evidence produces by driver/rider injury sustained during traffic accident show that, the majority of drivers/riders (75.38%) had no injury during a traffic accident. This development could be attributed to the modern technology which seeks to protect the driver by installing air bags and other safety materials which help safeguard and enhanced drivers’ safety. This suggests that drivers/riders are safer when accidents happen than occupants or pedestrians. This support a study by Pedley et al., (2003), which concluded that front seat passengers are at increased risk of injury of drivers in real road traffic accidents because they wear a seat belt during driving. Another possible explanation could be that they control the steering wheel and most often realize the incident before it happened which make them less vulnerable. It is arguable from this analysis that, since drivers are always safe during RTA, some precautionary measures which can help to avoid most of the avoidable accidents are not being observed properly.

The data shows that, a higher number of crashes are recorded on roads classified as good for transportation. This is very disturbing since good roads are expected to reduce the occurrence of traffic accidents. The biggest challenge as it has always been with the developing countries is enforcing laws which sometimes results in disobediences among road users. The consequences of poor enforcement are very grim and increasingly apparent since more violations such as reckless driving, noncompliance of traffic signals and animals finding their way onto the roads are often reported in the media. The failure in the application of reasonable force and legal sanctions to conduct that infringes the legitimate societal values, encourages lawlessness and insecurity. It is not common to find police or highway patrol monitoring and checking vehicle speed and whether drivers are following the lay-down norms. A democratic society is ruled by law and therefore, the law enforcing agencies must ensure full compliance of the law to the latter without fear or favour to control the situation.

It is fundamentally very important to acknowledge that seeing and being seen is needed to guarantee safety of all road users. The weather is always dynamic and one cannot deny the fact that high winds, rain, drizzle, fog and temperature in its extreme form can affect driver capabilities, traffic flow and vehicle performance. The analysis shows that traffic accidents often occurr under good weather conditions which contradicts the (WHO, 2004), world report on road traffic injury prevention, which says that poor visibility of both pedestrians and vehicles are very cumbersome, especially in the low and middle income countries and that road crashes among road users are mostly influenced by poor visibility. Again, this could be associated with lack orderliness and patience of all road users. This could be avoided if extra care is always observed. Being cautious means that drivers/riders must at all times check their left, right, and then again left side when proceeding through an intersection to ensure that the road is free from any obstruction.

## 5. Conclusion

The analysis has showcased the possible influences that come into play in causing a traffic accident in the country which is very important in road safety prevention programs and strategies. It is clear from the study that:

- Young drivers/riders (21-40) are more involved in causing RTA in the country.
- RTA is mostly not influenced by alcohol intake.
- Drivers/riders must be blamed in RTA occurances rather than vehicles.
- Less injury is sustained by drivers/riders during causalities.
- Good roads produce high road traffic accidents.
- A majority of RTA occurs during clear weather conditions.

It is obvious that RTA is very high and a pose a danger to the development of the country. There is a need for the road safety lead agencies to step up campaign strategies and specifically target these factors which have interconnectivity with human attitudes and a threat to innocent lives and properties. The policy makers must follow successful stories and approach their road safety campaign by engaging in a public education through the electronic and print media, transport stations and other social gatherings, to draw attention to the dangers and safety practices on the road. There should be periodic refresher courses for younger drivers/riders on traffic law to bring to bear the adherence of good driving/riding principles and attitudes. The results should pre-empt the law enforcement agencies to adopt a minimum force in the application of traffic law and thoroughly check the drivers’ actions at any given time. Stakeholders must also regularize and enforce the “Decade of action for road safety” adopted by the United Nations and the WHO to augment the road traffic act to achieve a considerable decrease of crashes in the country.

However, the study could not show the male-female drivers/riders causalities due to data availability. It is significant to establish the trend that exists between male-female drivers/riders traffic accident rate for safety planning and implementation purposes. Also, specific day and time during RTA was not analysed to substantiate its impact because of the above-mentioned reason which is important in determining traffic crashes. It is important that further research is conducted to look at all these factors which would inform stakeholders in the transport industry how to plan and adopt a holistic approach to eliminate this nuisance on the road.
